# Magnetic Order with Fractionalized Excitations in Pyrochlore Magnets with Strong Spin-Orbit Coupling

**DOI:** 10.1038/s41598-019-47517-6

**Published:** 2019-07-29

**Authors:** Li Ern Chern, Yong Baek Kim

**Affiliations:** 1 Department of Physics, University of Toronto, Toronto, Ontario M5S 1A7 Canada; 2 Canadian Institute for Advanced Research/Quantum Materials Program, Toronto, Ontario M5G 1Z8 Canada; 3 School of Physics, Korea Institute for Advanced Study, Seoul, 130-722 Korea

**Keywords:** Magnetic properties and materials, Electronic properties and materials

## Abstract

A recent inelastic neutron scattering experiment on the pyrochlore magnet Yb_2_Ti_2_O_7_ uncovers an unusual scattering continuum in the spin excitation spectrum despite the splayed ferromagnetic order in the ground state. While there exist well defined spin wave excitations at high magnetic fields, the one magnon modes and the two magnon continuum start to strongly overlap upon decreasing the field, and eventually they become the scattering continuum at zero field. Motivated by these observations, we investigate the possible emergence of a magnetically ordered ground state with fractionalized excitations in the spin model with the exchange parameters determined from two previous experiments. Using the fermionic parton mean field theory, we show that the magnetically ordered state with fractionalized excitations can arise as a stable mean field ground state in the presence of sufficiently strong quantum fluctuations. The spin excitation spectrum in such a ground state is computed and shown to have the scattering continuum. Upon increasing the field, the fractionalized magnetically ordered state is suppressed, and is eventually replaced by the conventional magnetically ordered phase at high fields, which is consistent with the experimental data. We discuss further implications of these results to the experiments and possible improvements on the theoretical analysis.

## Introduction

The family of rare earth pyrochlore compounds is the exemplar of three dimensional frustrated magnets that offer tremendous opportunities for the discovery of exotic phases of matter. For instance, one of the most celebrated emergent phenomena in condensed matter physics is the identification of low energy excitations as effective magnetic monopoles^1–4^ in the classical spin ice materials Ho_2_Ti_2_O_7_ and Dy_2_Ti_2_O_7_, where the rare earth ion carries large magnetic moment *μ* ≈ 10 *μ*_B_ subjected to strong local Ising anisotropy. Many other pyrochlore compounds, such as Yb_2_Ti_2_O_7_, Yb_2_Sn_2_O_7_, Tb_2_Ti_2_O_7_, and Pr_2_Zr_2_O_7_ ^5^, to name a few, are characterized by strong quantum fluctuations and complex exchange interactions. They are less understood and currently still under intense experimental and theoretical investigations. Among the exciting prospects is the realization of the long-sought-after quantum spin liquid state^5–10^, which is devoid of magnetic order down to very low temperatures while exhibiting long range entanglement and fractionalized excitations, in these materials.

In Yb_2_Ti_2_O_7_, the low energy degrees of freedom of each Yb^3+^ ion is described by a Kramers doublet well separated from the first excited crystal field states^11–13^, so that the system can be treated as a pyrochlore array of pseudospin-1/2 moments. A number of experiments^14–19^ have identified the splayed/noncollinear ferromagnetic order, where a net magnetization develops through canted spins, as the ground state of Yb_2_Ti_2_O_7_. Yet a recent inelastic neutron scattering experiment^20^ on Yb_2_Ti_2_O_7_ revealed some remarkably unconventional features in the magnetic ground state. While sharp one magnon modes and a two magnon continuum are well separated at high magnetic fields, they overlap with each other upon lowering the field, which leads to strong renormalization of the spin wave dispersions. As the field approaches zero, well defined spin wave dispersions can no longer be observed over a large region in the Brillouin zone, whereas a broad scattering continuum appears. This is interpreted in ref.^20^ as a consequence of one magnon decaying into two magnons, and their interaction is so strong that the linear spin wave theory is no longer an adequate description of the system.

The breakdown of magnons suggests the presence of strong quantum fluctuations despite the magnetic order in the ground state^21,22^. Since the scattering continuum seen in the experiment is reminiscent of the two spinon continuum in a quantum spin liquid, it may be useful to start from the extreme quantum limit or the spinon representation of the spin exchange interactions. The magnetically ordered state is obtained via confinement of spinons in the underlying spin liquid state. If the magnetically ordered state is on the verge of making a phase transition to a nearby spin liquid state, the confinement energy scale may be very small. It is then conceivable that the two spinon continuum could be seen above the small confinement energy scale, providing an alternative description of the scattering continuum seen in the experiment. With this picture in mind, we investigate in this work the possibility of a quantum spin liquid coexisting with a magnetic order, where the ground state is magnetically ordered, but the deconfined spinons exist as elementary excitations. Such a coexisting phase is possible in three dimensions and some two dimensional systems^8,23–26^, in which the deconfined spinons are inherited from some parent spin liquid states. There may be a transition from the coexisting phase to a confining phase with conventional magnetic order upon tuning the parameters of the model.

**Figure 1:**
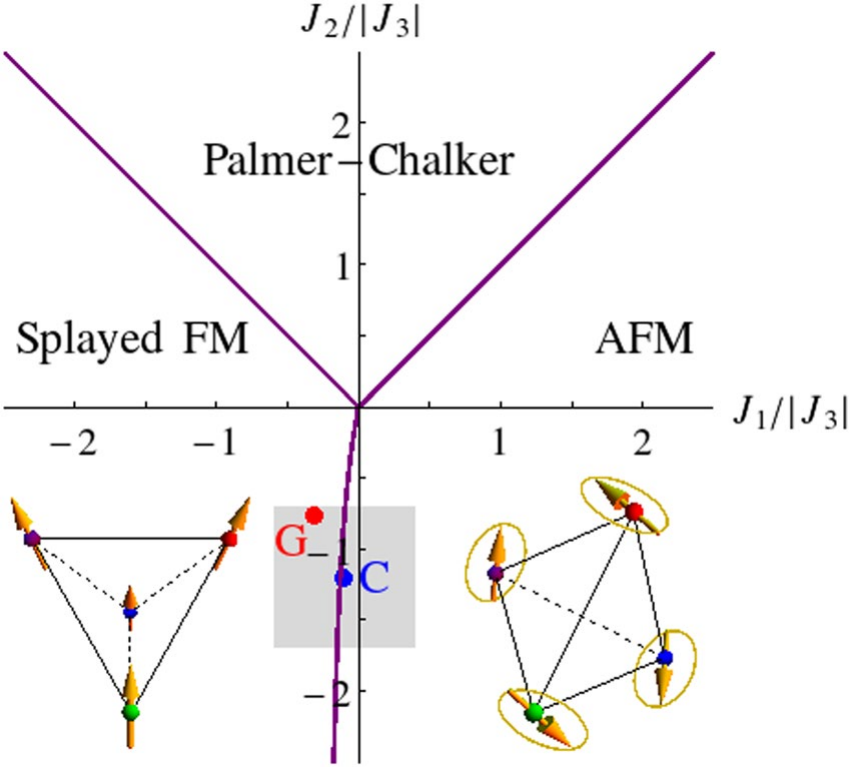
The classical phase diagram of the pyrochlore lattice in the *J*_1_ − *J*_2_ phase space (with *J*_3_ = −1, and *J*_4_ = 0 for simplicity) reported in ref.^35^. The location of Gaulin and Coldea parametrizations, (*J*_1_, *J*_2_) = (−0.31, −0.76) and (−0.1, −1.2), are indicated. They are very close to the phase boundary between the splayed ferromagnetic order, where a finite net magnetization develops along one of the cubic axes through canted spins, and the antiferromagnetic order, which is a one dimensional manifold of states (indicated by the circular loops) with zero net magnetization. It is shown in ref.^35^ that the nearest neighbor bilinear spin model (1) on the pyrochlore lattice admits only **q** = **0** orderings, i.e. all the possible symmetry breaking patterns are invariant under a Bravais lattice translation. It is thus sufficient to know the arrangement of spins on a tetrahedral unit.

We apply the complex fermion mean field theory^27–30^ to the nearest neighbor *S* = 1/2 Hamiltonian

1 $$H=\sum _{\langle ij\rangle }\,\sum _{\mu \nu }\,{S}_{i}^{\mu }{J}_{ij}^{\mu \nu }{S}_{j}^{\nu }-\sum _{i}\,\sum _{\mu \nu }\,{\mu }_{{\rm{B}}}{B}^{\mu }{g}^{\mu \nu }{S}_{i}^{\nu }$$

on the pyrochlore lattice, which in general contains four independent exchange parameters *J*_1_, *J*_2_, *J*_3_, and *J*_4_ (defined in the *global* coordinates)^31^. We list Gaulin^31^ and Coldea^20^ parametrizations of Yb_2_Ti_2_O_7_, which are obtained from spin wave analysis of the inelastic neutron scattering data at high magnetic fields, in Table 1. From the second row, we see that *J*_4_ is negligible in Gaulin parametrization, though comparable to *J*_1_ in Coldea parametrization. Still, it is one order of magnitude less than *J*_2_ and *J*_3_ in both cases. Therefore, to decrease the level of complexity we set *J*_4_ = 0, so that the spin Hamiltonian reduces to the *JK*Γ model. In the absence of the magnetic field **B**,

2 $$H=\sum _{\lambda =x,y,z}\,\sum _{\langle ij\rangle \in \lambda }\,J{{\bf{S}}}_{i}\cdot {{\bf{S}}}_{j}+K{S}_{i}^{\lambda }{S}_{j}^{\lambda }\pm {\rm{\Gamma }}({S}_{i}^{\mu }{S}_{j}^{\nu }+{S}_{i}^{\nu }{S}_{j}^{\mu })$$

where *J* = *J*_1_ is the Heisenberg interaction, *K* = *J*_2_ − *J*_1_ the Kitaev interaction, Γ = *J*_3_ the symmetric anisotropic exchange interaction, (*λ*, *μ*, *ν*) is a cyclic permutation of (*x*, *y*, *z*). The sign of the Γ coupling depends on the bond orientation. The spatially anisotropic exchange interactions *K* and Γ are known to cause strong magnetic frustration, for instance the pure Kitaev model on the honeycomb lattice supports an exactly soluble quantum spin liquid ground state^32^. Classically, it means that there exist degenerate ground states so that the system has difficulty in selecting one of them. From Table 1, *K* and Γ are manifestly the dominant exchange interactions in Yb_2_Ti_2_O_7_. It thus becomes clear why the system is so close to the classical phase boundary between two competing magnetically ordered phases, the splayed ferromagnetic state (abbrieviated as FM) and an antiferromagnetic state (abbreviated as AFM) with a *U*(1) symmetry, as shown in Fig. 1. We notice that Coldea parametrization falls into the AFM phase, but it is really an artifact of the simplification *J*_4_ = 0. This happens because the full parametrization, while sitting on the FM side, is extremely close to the FM/AFM boundary. Nevertheless, we will see later that a small magnetic field stabilizes the FM phase at the simplified Coldea parametrization. Also, these parametrizations should be taken as useful references but not exact values of the exchange interactions. Hence we will consider an extended neighborhood of Gaulin and Coldea parametrizations in the phase space rather than just taking two isolated points. The real parametrization of the material could be somewhere in this extended region.

**Table 1:** Gaulin and Coldea parametrizations in the local (*J*_*zz*_, *J*_±_, *J*_±±_, *J*_*z*±_) and global (*J*_1_, *J*_2_, *J*_3_, *J*_4_) coordinates, and in the form of standard exchanges (*J*, *K*, Γ, *D*). Energy is in units of meV.

	Gaulin	Coldea
local	(0.17, 0.05, 0.05, −0.14)	(0.026, 0.074, 0.048, −0.159)
global	(−0.09, −0.22, −0.29, 0.01)	(−0.028, −0.326, −0.272,0.049)
standard	(−0.09, −0.13, −0.29, 0.01)	(−0.028, −0.298, −0.272, 0.049)

We envision that a new quantum ground state such as the coexisting phase or a pure spin liquid phase may emerge near the classical phase boundary. We examine the conditions under which the fractionalized magnetically ordered phase emerges as a stable mean field ground state and find that, as discussed later, it appears only when quantum fluctuations are sufficiently strong. We present the *J*_1_ − *J*_2_ phase diagram (with *J*_3_ = −1 fixed) in the vicinity of Gaulin and Coldea parametrizations (the shaded region in Fig. 1). Our results demonstrate that the low lying excitation continuum observed in the recent inelastic neutron scattering experiment^20^ on Yb_2_Ti_2_O_7_ at weak magnetic fields may be attributed to deconfined spinons in the fractionalized magnetically ordered phase. The disappearance of the spin liquid/coexisting phase under sufficiently strong magnetic fields, which signals the complete confinement of spinons, is also consistent with the absence of such continuum and the presence of sharp magnon modes at high magnetic fields in the experiment. We establish the splayed ferromagnetic state with deconfined spinons as an alternative account of the experimental findings at the qualitative level. The coexistence of scattering continuum and magnetic order also occurs in other related rare earth pyrochlore compounds, for example Yb_2_Ge_2_O_7_ which orders antiferromagnetically but shows diffusive spin excitations similar to that of Yb_2_Ti_2_O_7_ ^33^. Our result may thus provide a useful starting point for a unified theoretical description of such a phenomenon in these systems.

## Results

### Complex fermion mean field theory

The spin operator is first represented in terms of fermionic spinon creation and annihilation operators as

3 $${{\bf{S}}}_{i}=\frac{1}{2}\sum _{\alpha \beta }\,{f}_{i\alpha }^{\dagger }{\sigma }_{\alpha \beta }{f}_{i\beta },$$

so that the Hamiltonian (1) is quartic in these spinon operators. Next, we rewrite the Hamiltonian as a summation of bilinears of various order parameters, and perform a mean field decoupling (see Supplementary Sec. S3 for more details). The mean field theory is then solved self consistently. On the one hand, we have the spinon pairing and hopping channels that signify the quantum spin liquid state^29^,

4a $${\hat{\chi }}_{ij}=\sum _{\alpha }\,{f}_{i\alpha }^{\dagger }{f}_{j\alpha },$$

4b $${\hat{{\rm{\Delta }}}}_{ij}=\sum _{\alpha \beta }\,{f}_{i\alpha }{[i{\sigma }^{y}]}_{\alpha \beta }{f}_{j\beta },$$

4c $${\hat{E}}_{ij}^{\mu }=\sum _{\alpha \beta }\,{f}_{i\alpha }^{\dagger }{\sigma }_{\alpha \beta }^{\mu }{f}_{j\beta },$$

4d $${\hat{D}}_{ij}^{\mu }=\sum _{\alpha \beta }\,{f}_{i\alpha }{[i{\sigma }^{y}{\sigma }^{\mu }]}_{\alpha \beta }{f}_{j\beta }.$$

A specific choice of the respective mean field parameters {*χ*_*ij*_, Δ_*ij*_, **E**_*ij*_, **D**_*ij*_} is called an ansatz for the spin liquid. We consider the ${{\mathbb{Z}}}_{2}$ uniform ansatz (abbreviated as ${{\mathbb{Z}}}_{2}{\rm{U}}$) and the *U*(1) monopole flux ansatz^34^ (abbrieviated as *U*(1)M) as the possible spin liquid ground states (see Supplementary Sec. S4 in for more details). On the other hand, we have the magnetic channels that signify the FM and AFM orders^35^,

5a $${{\bf{m}}}_{{{\rm{T}}}_{1,{\rm{A}}^{\prime} }}=\frac{1}{2}\,\cos \,{\theta }_{{{\rm{T}}}_{1}}(\begin{array}{c}{S}_{0}^{x}+{S}_{1}^{x}+{S}_{2}^{x}+{S}_{3}^{x}\\ {S}_{0}^{y}+{S}_{1}^{y}+{S}_{2}^{y}+{S}_{3}^{y}\\ {S}_{0}^{z}+{S}_{1}^{z}+{S}_{2}^{z}+{S}_{3}^{z}\end{array})+\frac{1}{2\sqrt{2}}\,\sin \,{\theta }_{{{\rm{T}}}_{1}}(\begin{array}{c}{S}_{0}^{y}+{S}_{0}^{z}-{S}_{1}^{y}-{S}_{1}^{z}-{S}_{2}^{y}+{S}_{2}^{z}+{S}_{3}^{y}-{S}_{3}^{z}\\ {S}_{0}^{z}+{S}_{0}^{x}+{S}_{1}^{z}-{S}_{1}^{x}-{S}_{2}^{z}-{S}_{2}^{x}-{S}_{3}^{z}+{S}_{3}^{x}\\ {S}_{0}^{x}+{S}_{0}^{y}-{S}_{1}^{x}+{S}_{1}^{y}+{S}_{2}^{x}-{S}_{2}^{y}-{S}_{3}^{x}-{S}_{3}^{y}\end{array}),$$

5b $${{\bf{m}}}_{{\rm{E}}}=(\begin{array}{c}\frac{1}{2\sqrt{6}}(\,-\,2{S}_{0}^{x}+{S}_{0}^{y}+{S}_{0}^{z}-2{S}_{1}^{x}-{S}_{1}^{y}-{S}_{1}^{z}+2{S}_{2}^{x}+{S}_{2}^{y}-{S}_{2}^{z}+2{S}_{3}^{x}-{S}_{3}^{y}+{S}_{3}^{z})\\ \frac{1}{2\sqrt{2}}(\,-\,{S}_{0}^{y}+{S}_{0}^{z}+{S}_{1}^{y}-{S}_{1}^{z}-{S}_{2}^{y}-{S}_{2}^{z}+{S}_{3}^{y}+{S}_{3}^{z})\end{array}),$$

where the subscripts 0, 1, 2, and 3 denote the four sublattices of a tetrahedral unit in the pyrochlore lattice, and ${\theta }_{{{\rm{T}}}_{1}}=1/2\times {\tan }^{-1}[\sqrt{8}{J}_{3}/(2{J}_{1}+2{J}_{2}+{J}_{3}-2{J}_{4})]$. There are other classical phases than the FM and AFM orders, for instance the Palmer-Chalker phase shown in Fig. 1. However, these other classical phases are energetically unfavorable in the neighborhood of the parametrizations of Yb_2_Ti_2_O_7_, so their respective order parameters will vanish if we formally include them. Here we simply set them to be zero. Interested readers can refer to Table III and V in ref.^35^ for the complete set of magnetic order parameters. Such a strategy of incorporating both spin liquid and magnetic order channels within a single framework using complex fermion mean field theory has been similarly applied in refs^25,26^.

**Figure 2:**
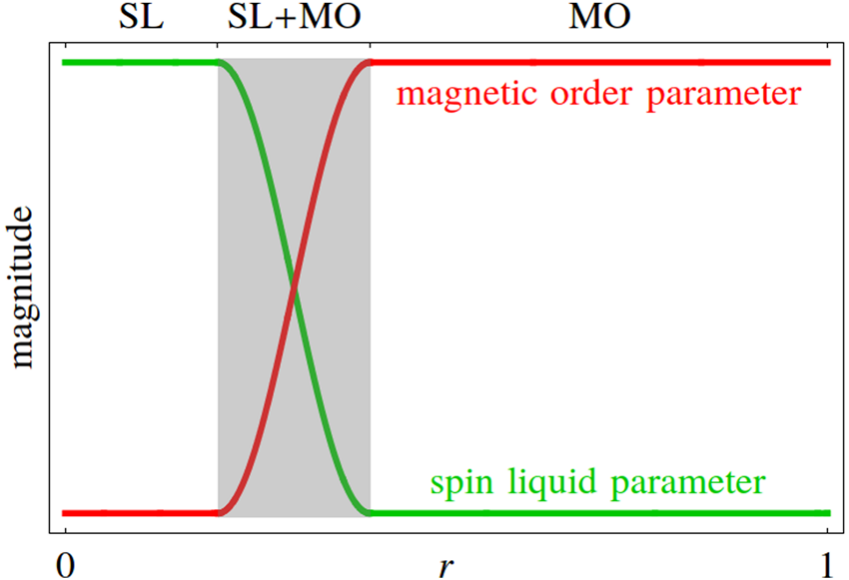
Schematic behavior of the system described by our mean field theory. The spin liquid channels and the magnetic order channels are weighted by the factors 1 − *r* and *r* respectively. For small (large) values of the weighting factor *r*, only the spin liquid (magnetic order) parameters are finite, and the system is in a pure quantum spin liquid (magnetically ordered) phase. For intermediate values of *r* (the shaded region), both the spin liquid and magnetic order parameters are finite, and the system is in a coexisting or fractionalized magnetically ordered phase.

Since the original spin Hamiltonian (1) can be expressed solely in terms of the spin liquid channels *or* the magnetic order channels, an ambiguity arises when we try to incorporate both types of channels into a single Hamiltonian. A relative weight *r* ∈ [0, 1] is thus introduced such that the spin liquid component is multiplied by a factor of 1 − *r*, while the magnetic order component by *r*. *r* = 0 (*r* = 1) then correponds to the quantum (classical) limit and yields a pure spin liquid (magnetic order) description. Physically speaking, *r* is a parameter that controls the strength of quantum fluctuations in the system, with smaller *r* indicating stronger quantum fluctuations. The value of *r* is not fixed at the mean field level. We find that there exists a window of intermediate values of *r* for which the coexisting phase, where both spin liquid and magnetic order parameters are finite (see Fig. 2), is stabilized in the vicinity of the experimentally determined parametrizations. With several choices of the weighting factor *r* and the magnetic field *B*_*z*_, we plot the *J*_1_ − *J*_2_ phase diagram (with *J*_3_ = −1 fixed) around Gaulin and Coldea parametrizations.

### Phase diagram

**Figure 3:**
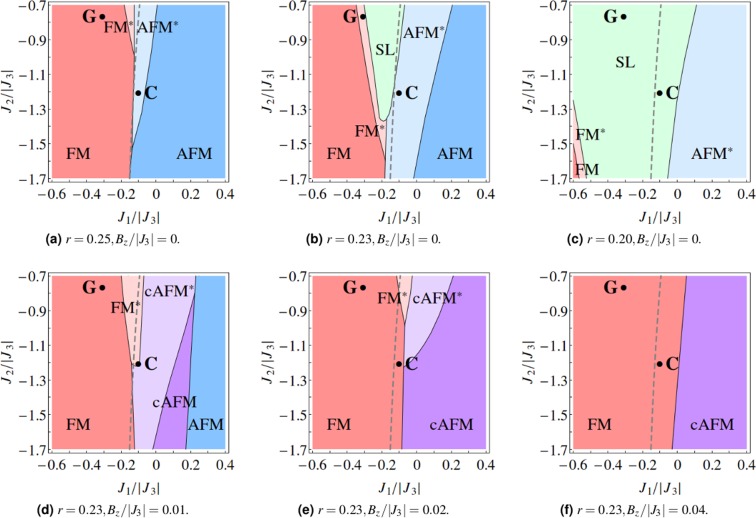
The *J*_1_ − *J*_2_ phase diagrams (with *J*_3_ = −1) with the ${{\mathbb{Z}}}_{2}{\rm{U}}$ ansatz. The gray dashed line represents the classical phase boundary between the FM and AFM orders (see Fig. 1). The phase diagrams at zero magnetic field, with weighting factors (**a**) *r* = 0.25, (**b**) *r* = 0.23, and (**c**) *r* = 0.20. The area with deconfined spinons expands as *r* decreases due to the suppression of classical order. Choosing a representative weighting factor *r* = 0.23, we apply magnetic fields (**d**) *B*_*z*_ = 0.01|*J*_3_|, (**e**) *B*_*z*_ = 0.02|*J*_3_|, and (**f**) *B*_*z*_ = 0.04|*J*_3_| along one of the cubic axes. The area with deconfined spinons shrinks and eventually disappears with increasing field. The meaning of the various labels can be found in the main text.

For the ${{\mathbb{Z}}}_{2}{\rm{U}}$ ansatz, when the weighting factor *r* is decreased to about 0.25, we can stabilize the coexisting phase over a finite area in the *J*_1_ − *J*_2_ phase space (see Fig. 3a). We label this phase by FM^*^ or AFM^*^ depending on which magnetic order parameter is turned on, which means ‘a magnetically ordered state with fractionalized excitations/deconfined spinons’. As *r* is further decreased, for instance to 0.23 and 0.20, the classical ordering is further suppressed, the phase region with deconfined spinons expands, and a pure spin liquid phase, where all the magnetic order parameters converge to zero, emerges (see Fig. 3b,c). We calculate the magnitude of the expectation value of the spin operator $S\equiv |\langle \hat{{\bf{S}}}\rangle |$, and compare it to the normalization in the classical limit *S*_0_≡|**S**_*i*_| = 1/2. The ratio *S*/*S*_0_ is unity (zero) when the system is in the pure magnetically ordered state (pure spin liquid state), while 0 < *S*/*S*_0_ < 1 in the fractionalized magnetically ordered state. The latter can be interpreted as the reduction of ordered moments due to quantum fluctuations.

**Figure 4:**
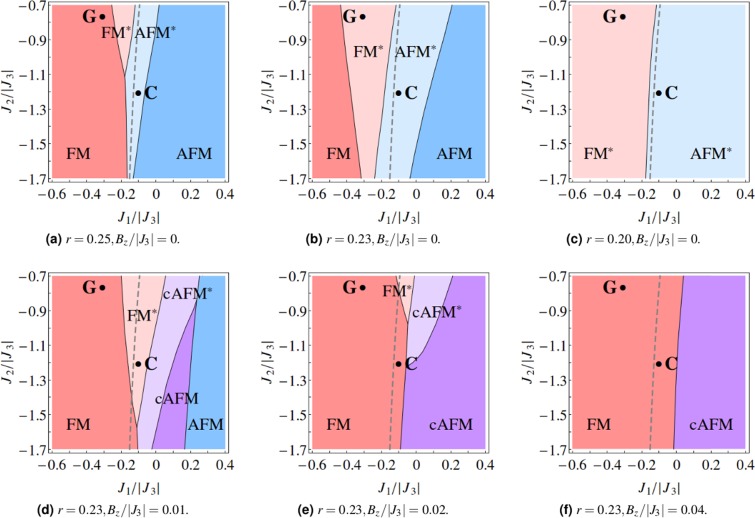
The *J*_1_ − *J*_2_ phase diagrams (setting *J*_3_ = −1) with the *U*(1)M ansatz, at various weighting factors *r* and magnetic fields *B*_*z*_ studied above. The main difference between the *U*(1)M and ${{\mathbb{Z}}}_{2}{\rm{U}}$ ansatzes is that no pure spin liquid state appears in the phase diagram, as the magnetic order parameter always converges to some finite number, although it can be as small as $\lesssim 0.01$ of its classical value (compare (**a–c**) here to Fig. 3a–c). The qualitative features which remain the same are that the area with deconfined spinons expands as *r* decreases due to the suppression of classical order, and shrinks with increasing magnetic field *B*_*z*_.

For the *U*(1)M ansatz, we can similarly obtain the coexisting phases FM^*^ and AFM^*^ at *r* ~ 0.25 (see Fig. 4a). However, in these phases, *S*/*S*_0_ ~ 0.01, leading to a small but finite magnetic order parameter and indicating the system is highly quantum. In contrast, for the ${{\mathbb{Z}}}_{2}{\rm{U}}$ ansatz, *S*/*S*_0_ is usually of the order of 0.1 in the coexisting phase. Interestingly, decreasing *r* further to 0.23 and 0.20, the area in the phase space with deconfined spinons expands (see Fig. 4b,c), but always with a finite magnetic order parameter, whose magnitude can be as small as $\lesssim 0.01$ of the classical value. Strictly speaking, no pure spin liquid phase is obtained in this case, but one can say that the coexisting phase obtained with the *U*(1)M ansatz is almost a pure spin liquid due to extremely small magnetic order parameter.

We pick a representative value of the weighting factor *r* = 0.23 and investigate the evolution of phase diagram with the application of magnetic field along one of the cubic axes (in the *z* direction, say). The FM phase is energetically favored under such a field. The *g* tensor in (1) takes the form diag(*g*_*xy*_, *g*_*xy*_, *g*_*z*_) in the *local* coordinates. We fix the *g* factors *g*_*xy*_ = 4.2 and *g*_*z*_ = 2.0, based on the reported values of (*g*_*xy*_, *g*_*z*_) = (4.27, 1.79) and (4.17, 2.14) in refs^11,20^ respectively. We also absorb the Bohr magneton into the magnetic field, *μ*_B_**B** → **B**, so that the latter has the same unit as energy. With increasing field strength, we observe that the phase region with deconfined spinons shrinks, while that of FM grows and crosses the classical phase boundary at zero field (see Figs 3d,e or 4d,e). It is also possible to obtain a solution where both the FM and AFM order parameters are finite and comparable, on top of which the spin liquid parameters may be zero or finite. The resulting phase is typically a canted AFM state with a finite FM moment, so we label it by cAFM or cAFM^*^. These phases are absent in the zero field limit. When the FM and AFM order parameter have about the same magnitude (e.g. $|{{\bf{m}}}_{{{\rm{T}}}_{1,A\text{'}}}| \sim 0.7$ and |**m**_E_| ~ 0.7), the precise ordering pattern of spins in the cAFM phase can be visualized by evaluating and plotting the expectation value of the spin operator $\langle {\hat{{\bf{S}}}}_{i}\rangle$. Otherwise, if one of the FM and AFM order parameters is much larger than the other (e.g. $|{{\bf{m}}}_{{{\rm{T}}}_{1,A\text{'}}}| \sim 0.9$ and |**m**_E_| ~ 0.1), then the spin configuration of the cAFM phase will of course resemble the dominant order. Eventually, when the field strength is sufficiently large, the phase region with deconfined spinons vanishes entirely and the system becomes classical in the neighborhood of Gaulin and Coldea parametrizations (see Figs 3f or 4f).

### Local and global minima

We pick a representative value of the weighting factor *r* = 0.23 to extract some qualitative features of the mean field solutions at the Gaulin and Coldea parametrizations. In the zero field limit, with the ${{\mathbb{Z}}}_{2}{\rm{U}}$ ansatz, these parametrizations are in the fractionalized magnetically ordered phases, but are located very close to the pure spin liquid phase (see Fig. 3b). On the other hand, with the *U*(1)M ansatz, the magnetic order is very weak, i.e. *S*/*S*_0_ is very small, in the FM^*^ and AFM^*^ phases, at Gaulin and Coldea parametrizations respectively (see Fig. 4b and Supplementary Tables S4 and S5). These suggest that a pure spin liquid phase is energetically competitive with the fractionalized magnetically ordered ground states.

Indeed, for the ${{\mathbb{Z}}}_{2}{\rm{U}}$ ansatz, we find that the pure spin liquid state is another convergent solution from the self consistent calculations, but it corresponds to a local minimum, in the zero field limit. We compare the energies of the two mean field solutions corresponding to the local and global minima, where the spin liquid ($S/{S}_{0}\ll 1$) and magnetic order (*S*/*S*_0_ ~ 1) dominate respectively. Similar comparison is made for the *U*(1)M ansatz, where the spin liquid dominant coexisting state is also the ground state at small enough fields. Details can be found in Supplementary Sec. S5. The energy difference between the local and global minima is quite small in the low field limit, but becomes significant as the magnetic field *B*_*z*_ increases in strength such that the FM state is more favorable. When the field strength is sufficiently large (*B*_*z*_ $\gtrsim$ 0.01|*J*_3_|), we can no longer get the FM^*^ phase, the self consistent calculation always yields the FM phase, and the system becomes fully classical. The coexisting solution for the Coldea parametrization is relatively more persistent with increasing field compared to the Gaulin parametrization.

### Spinon dispersion and dynamical spin structure factor

**Figure 5:**
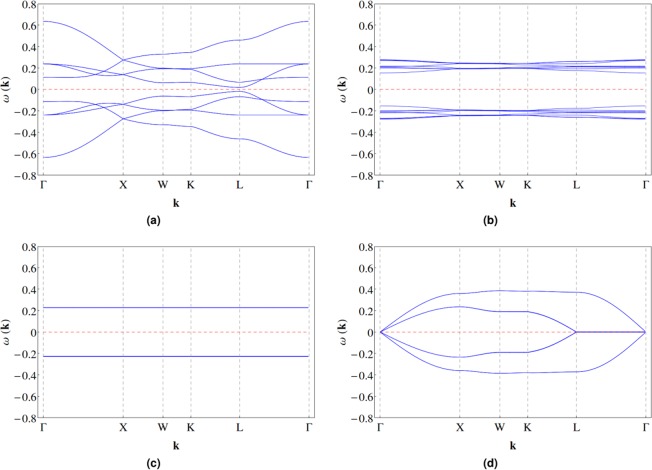
The spinon band structures of the various phases at Gaulin parametrization. (**a**) The pure spin liquid state with the ${{\mathbb{Z}}}_{2}{\rm{U}}$ ansatz. Each band is two fold degenerate. (**b**) The magnetic order dominant FM^*^ ground state with the ${{\mathbb{Z}}}_{2}{\rm{U}}$ ansatz. Each band is non-degenerate. In (**a**,**b**), the red dashed horizontal line indicates the zero level, above which the excitation spectrum of Bogoliubov quasiparticles of spinons lies. (**c**) The pure magnetically ordered (FM) state. Each band is four fold degenerate. (**d**) The pure spin liquid state with the *U*(1)M ansatz. Each band is two fold degenerate. In (**c**,**d**), the red dashed line indicates the Fermi level of spinons. Detailed discussion can be found in the main text.

With the mean field solutions, we can plot the spinon band structures of the various phases along some high symmetry directions in the Brillouin zone^36^. To illustrate the generic behavior, we consider the ${{\mathbb{Z}}}_{2}{\rm{U}}$ ansatz at Gaulin parametrization with *r* = 0.23. At zero field, the pure spin liquid state is a local minimum, and has a small gap of the order of 0.01|*J*_3_| (see Fig. 5a). The bands are two fold degenerate due to the presence of both inversion and time reserval symmetries. In comparison, the global minimum is a FM^*^ phase with dominant magnetic order (see Fig. 5b). The two fold degeneracy is lifted, the spinon dispersion is relatively flat, and the excitation gap is relatively large. At finite fields, the magnetic order parameter (spin liquid parameters) further increases (decreases), the bands becomes less dispersing and eventually completely flatten out as the system enters the pure magnetically ordered (FM) phase when the field is sufficiently large (see Fig. 5c). The flat bands are four fold degenerate (see Supplementary Sec. S6 for explanation).

On the other hand, for the *U*(1)M ansatz, the pure spin liquid state is gapless (see Fig. 5d). Although the ground state is a FM^*^ phase, it is very proximate to the pure spin liquid state as the magnetic order is very weak (see Supplementary Table S4 and the discussion above). The bands are two fold degenerate because, while the inversion and time reserval symmetries are broken separately, the combination of them is a symmetry^34^. When the magnetic order parameter becomes significant with increasing field, the band degeneracy is lifted, an excitation gap appears and grows, the bands becomes less dispersing and gradually flatten out. The corresponding figures are not shown here for brevity as these observations are qualitatively similar to the case with the ${{\mathbb{Z}}}_{2}{\rm{U}}$ ansatz.

Importantly, as long as the spin liquid parameters are not all zero, i.e. in the pure spin liquid phase or the coexisting phase, we will have dispersing spinon bands and thus a two spinon continuum, which is related to the dynamical spin structure factor,

6 $$S({\bf{k}},\omega )=\sum _{ij}\,{e}^{-i{\bf{k}}\cdot ({{\bf{r}}}_{i}-{{\bf{r}}}_{j})}\int {\rm{d}}t{e}^{i\omega t}\langle {{\bf{S}}}_{i}(t)\cdot {{\bf{S}}}_{j}(0)\rangle $$

**Figure 6:**
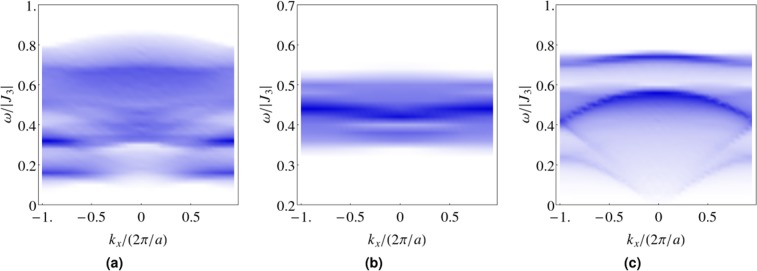
The dynamical spin structure factors at Gaulin parametrization in the zero field limit. (**a**) The pure spin liquid state with the ${{\mathbb{Z}}}_{2}{\rm{U}}$ ansatz, where *S*/*S*_0_ = 0, (**b**) the magnetic order dominant FM^*^ ground state with the ${{\mathbb{Z}}}_{2}{\rm{U}}$ ansatz, where *S*/*S*_0_ = 0.975, and (**c**) the spin liquid dominant FM^*^ ground state with the *U*(1)M ansatz, where *S*/*S*_0_ = 0.008. Darker regions indicate higher intensities. A broad (narrow) continuum is obtained when the spin liquid (magnetic order) parameters dominate.

We plot the dynamical spin structure factor for a few illustrative cases along the *k*_*x*_ direction. In the coexisting phase, the width of the continuum depends on the relative magnitude of the spin liquid parameters to the magnetic order parameters. If the spin liquid parameters dominate over the magnetic order parameters, i.e. the ratio *S*/*S*_0_ → 0 is small and the quantum effect is strong, then a broad continuum is obtained (see Fig. 6a,c). If the converse is true, i.e. *S*/*S*_0_ → 1 and the quantum effect is weak, then a narrow continuum is obtained (see Fig. 6b).

## Discussion

In summary, we have applied the complex fermion mean field theory to the *JK*Γ model of the *S* = 1/2 pyrochlore magnet. We have devised a Hamiltonian that includes both the spin liquid and magnetic order channels with a relative weight *r* between them. By decreasing *r* and hence increasing quantum fluctuations, we have found that the fractionalized magnetically ordered state can be stabilized in the vicinity of the parametrizations of Yb_2_Ti_2_O_7_. Such a coexisting phase, which has reduced ordered moments and deconfined spinons, may explain the peculiar low lying excitation continuum observed in the recent inelastic neutron scattering experiment. Furthermore, in the coexisting phase, given a particular spin liquid ansatz, if the spin liquid component is dominant, then naturally the two spinon continuum will look similar regardless of the underlying magnetic order. Our result may thus account for the resemblance of the diffusive spin excitation spectrum of Yb_2_Ti_2_O_7_ (a splayed ferromagnet) to that of Yb_2_Ge_2_O_7_ (an antiferromagnet) observed in inelastic neutron scattering measurements^33^, assuming the low energy excitations in these two different systems originates from the same quantum spin liquid state.

At this stage, there is no systematic way to decide which value of *r* should be used within a mean field theory, but it may be determined dynamically if we go beyond the mean field theory. Nevertheless, we believe that some intermediate values of *r* correspond to the physical case as significant quantum fluctuations must be present in the ground state of the spin model with Gaulin and Coldea parametrizations. It would be great if there is a way to estimate the appropriate value of *r* with an analysis similar to the application of the Gutzwiller approximation in the *tJ* model, which leads to renormalization of the hopping integral *t* and the Heisenberg interaction *J*^37^.

An analogy can be drawn between our approach and the method of Schwinger boson mean field theory in refs^38–40^. In the latter, the original *SU*(2) Heisenberg antiferromagnet is treated with a large *N* expansion technique by introducing 2*N* flavors of bosonic spinons *b*_*iα*_, which transform under the symplectic group *Sp*(*N*), at each site *i*. For the smallest possible value of *N* = 1, *Sp*(1) is isomorphic to *SU*(2). It can be shown that the number of bosonic spinons per site (i.e. the density), ${n}_{b}\equiv \sum _{\alpha }\,{b}_{i\alpha }^{\dagger }{b}_{i\alpha }$, is related to the spin *S* by *n*_*b*_ = 2*S* for *SU*(2). For arbitrary *N*, the parameter *κ* ≡ *n*_*b*_/*N* is taken as the generalization of the spin *S*. By varying *κ*, one is able to access the magnetically ordered phase (*κ* → ∞, large “spin”) and the quantum spin liquid phase (*κ* → 0, small “spin”) within a single Hamiltonian. In other words, the choice of *κ* reflects the extent of quantum fluctuations such that large (small) values of *κ* correspond to the classical (quantum) limit. The weighting factor *r* in our present work can therefore be viewed as playing the same role as that of *κ* in Schwinger boson mean field theory.

We take the point of view that the experimentally observed scattering continuum at low magnetic fields and the two magnon continuum at high magnetic fields have different origins. At high fields, the system is magnetically ordered, and its behavior can be well accounted for by the linear spin wave theory. Sharp one magnon modes exist, and the two magnon continuum results from exciting pairs of these magnons. Such a two magnon continuum is naturally well separated in energy from the one magnon modes. At low fields, however, we interpret the continuum as coming from two spinon excitations, which would overlap with and strongly renormalize the magnon dispersion. In this sense, the two spinon continuum replaces the two magnon continuum at low magnetic fields. If one insists on describing the continuum in terms of spin wave theory, it would be necessary to go well beyond the linear spin wave theory and take into account the decay into multi-magnon excitations, not just the two magnon continuum.

We consider here only two quantum spin liquid ansatzes, the ${{\mathbb{Z}}}_{2}$ uniform and *U*(1) monopole flux states, which are allowed by the projective symmetry group^27^ (PSG) of the pyrochlore lattice. Certainly, there are many other competing spin liquid states that may also permit a coexisting magnetic order. In order to carry out a more systematic investigation, one will have to classify all the possible fermionic spin liquid states on the pyrochlore lattice. Future work on this issue will be desirable for a more complete analysis of the possible spin liquid and fractionalized magnetically ordered phases in Yb_2_Ti_2_O_7_.

## Methods

### Mean field self consistent equations

The mean field theory is solved by minimizing the Hamiltonian ${H}^{{\rm{MF}}}({\chi }_{ij},{{\rm{\Delta }}}_{ij},{{\bf{E}}}_{ij},{{\bf{D}}}_{ij},{{\bf{m}}}_{{{\rm{T}}}_{1,A\text{'}}},{{\bf{m}}}_{{\rm{E}}})$ with respect to the variational parameters, which yields the self consistent equations,

7 $$\begin{array}{rcl}\frac{\partial \langle {H}^{{\rm{MF}}}\rangle }{\partial O} & = & 0\iff O=\langle \hat{O}\rangle ,\\ O & = & {\chi }_{ij},{{\rm{\Delta }}}_{ij},{{\bf{E}}}_{ij},{{\bf{D}}}_{ij},{{\bf{m}}}_{{{\rm{T}}}_{1,A\text{'}}},{{\bf{m}}}_{{\rm{E}}},\end{array}$$

while Lagrange multipliers are introduced to enforce the single occupancy constraint

8 $$\sum _{\alpha }\,{f}_{i\alpha }^{\dagger }{f}_{i\alpha }=1$$

on average, as the Hilbert space is enlarged by the parton construction. The self consistent calculations are performed numerically in momentum space, through the Fourier transform

9 $${f}_{{\bf{k}},s,\alpha }=\frac{1}{\sqrt{N}}\sum _{{\bf{R}}}\,{f}_{{\bf{R}},s,\alpha }{e}^{-i{\bf{k}}\cdot {\bf{R}}},$$

where **R**, *s* and *α* label the unit cell, sublattice, and spin flavor respectively. We also acknowledge refs^41–46^.

## Supplementary information

Supplementary Materials

## Data Availability

All relevant data are available from the corresponding author upon reasonable request.
