# Supplementary material for: Magnetic Order with Fractionalized Excitations in Pyrochlore Magnets with Strong Spin-Orbit Coupling
Source: Sci Rep. 2019 Jul 29;9:10974. doi: 10.1038/s41598-019-47517-6 (PMC6662772; doi:10.1038/s41598-019-47517-6)
Supplement: Supplementary file 1 — Supplementary Materials [file 41598_2019_47517_MOESM1_ESM.pdf]

Supplementary Materials

Magnetic Order with Fractionalized Excitations in Pyrochlore Magnets with Strong Spin-Orbit Coupling

Li Ern Chern[1] and Yong Baek Kim[1,2,3]

[1]Department of Physics, University of Toronto, Toronto, Ontario M5S 1A7, Canada
[2]Canadian Institute for Advanced Research/Quantum Materials Program, Toronto, Ontario M5G 1Z8, Canada
[3]School of Physics, Korea Institute for Advanced Study, Seoul 130-722, Korea

S1 Structure and Symmetries of the Pyrochlore Lattice

The pyrochlore lattice is a three dimensional network of corner sharing tetrahedra (see Fig. S1). The underlying Bravais lattice is the face centered cubic (fcc) lattice, with four sites (or sublattices) per unit cell, which we label by $s = 0$, 1, 2, and 3. The space group of the pyrochlore lattice is Fd$\bar{3}$m[34], which consists of lattice translations and the full octahedral group O_h. The latter is most conveniently viewed as $T_d \times i$[35], where T_d is the full tetrahedral group of 24 elements, and i is the set containing identity e and inversion $\mathscr{I}$ about a site. The elements of T_d are best visualized by embedding the tetrahedron in a cube[43,44] as in Fig. S2:

- e: the identity;
- $8\,C_3$: rotation by $\pm 2\pi/3$ about one of the local $[111]$ axes (the directions along the center to the corners of the tetrahedron);
- $3\,C_2$: rotation by π about one of the cubic axes (x, y and z directions);
- $6\,S_4$: rotation by $\pm\pi/2$ about one of the cubic axes (e.g. x axis) followed by reflection across the plane perpendicular to that axis (e.g. yz plane);
- $6\,\sigma_{\mathrm{d}}$: reflection across one of the diagonal planes, which are perpendicular to the $[011]$, $[01\bar{1}]$, $[101]$, $[\bar{1}01]$, $[110]$, and $[1\bar{1}0]$ directions.

In Fig. S2, we have followed the choice of coordinates as in Ref.[31], such that the fcc Bravais lattice points are located at the centers of tetrahedra, and the sublattices $s = 0$, 1, 2, and 3 are displaced by $a/8\,(1,1,1)$, $a/8\,(1,-1,-1)$, $a/8\,(-1,1,-1)$, and $a/8\,(-1,-1,1)$ from the tetrahedral centers respectively, where a is the lattice constant of the conventional cubic cell (which contains four fcc Bravais lattice points). The inversion center is chosen to be the sublattice $s = 0$ in the unit cell at the origin $\mathbf{0}$.

S2 Local Coordinates, Spin Hamiltonian, and g Tensor

As discussed in the main text, in the *global* coordinates, the most general nearest neighbor spin Hamiltonian

$$H = \sum_{\langle ij \rangle} \sum_{\mu\nu} S_i^{\mu} J_{ij}^{\mu\nu} S_j^{\nu} \tag{S1}$$

allowed by the symmetries of pyrochlore lattice contains four independent exchange parameters J_1, J_2, J_3, and J_4[31]. J_1 is the Heisenberg interaction (J), $J_2 - J_1$ the Kitaev interaction (K), J_3 the symmetric anisotropic exchange interaction (Γ), and J_4 the Dzyaloshinskii Moriya interaction (D). For instance, the interaction between the spins at sublattice 0 and 1 is given by,

$$\begin{aligned} H_{01} &= \begin{pmatrix} S_0^x & S_0^y & S_0^z \end{pmatrix} \begin{pmatrix} J_2 & J_4 & J_4 \\ -J_4 & J_1 & J_3 \\ -J_4 & J_3 & J_1 \end{pmatrix} \begin{pmatrix} S_1^x \\ S_1^y \\ S_1^z \end{pmatrix} \\ &= J\mathbf{S}_0 \cdot \mathbf{S}_1 + K S_0^x S_1^x + \Gamma \left(S_0^y S_1^z + S_0^z S_1^y \right) + D \left(S_0^x S_1^y - S_0^y S_1^x + S_0^x S_1^z - S_0^z S_1^x \right). \end{aligned} \tag{S2}$$

It is clear that $\langle 01 \rangle$ is an x bond from the second equality. To relate the interactions on different bonds, we can use the C_3 rotations[31,35], for instance

$$\mathsf{J}_{02}^{\mathrm{global}} = O_{C_3^{[111]}} \mathsf{J}_{01}^{\mathrm{global}} O_{C_3^{[111]}}^{-1}. \tag{S3}$$

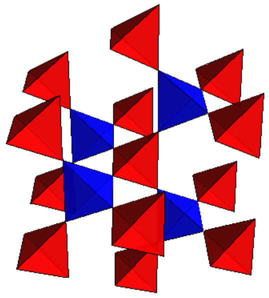

Figure S1. The sites of pyrochlore lattice form a three dimensional network of corner sharing tetrahedra. The up (down) tetrahedra are colored in red (blue). It is easy to see that each up (down) tetrahedron is surrounded by four down (up) tetrahedra. The underlying Bravais lattice is the face centered cubic (fcc) lattice with four sites (sublattices) per unit cell, which are located at the corners of the tetrahedra.

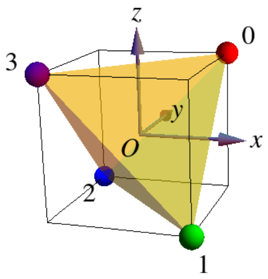

Figure S2. To visualize the full tetrahedral group T_d, we embed a tetrahedron in a cube and define a coordinate system with the cubic axes. The point group of the pyrochlore lattice is $O_h = T_d \times \{e, \mathscr{I}\}$, where e is the identity and $\mathscr{I}$ is inversion about a site.

The expression of $O_{C_3^{[111]}}$ can be found in (S29). We list all these interactions below for completeness.[35]

$$\mathsf{J}_{01}^{\text{global}} = \begin{pmatrix} J_2 & J_4 & J_4 \\ -J_4 & J_1 & J_3 \\ -J_4 & J_3 & J_1 \end{pmatrix}, \mathsf{J}_{02}^{\text{global}} = \begin{pmatrix} J_1 & -J_4 & J_3 \\ J_4 & J_2 & J_4 \\ J_3 & -J_4 & J_1 \end{pmatrix}, \mathsf{J}_{03}^{\text{global}} = \begin{pmatrix} J_1 & J_3 & -J_4 \\ J_3 & J_1 & -J_4 \\ J_4 & J_4 & J_2 \end{pmatrix},$$
$$\mathsf{J}_{12}^{\text{global}} = \begin{pmatrix} J_1 & -J_3 & J_4 \\ -J_3 & J_1 & -J_4 \\ -J_4 & J_4 & J_2 \end{pmatrix}, \mathsf{J}_{23}^{\text{global}} = \begin{pmatrix} J_2 & -J_4 & J_4 \\ J_4 & J_1 & -J_3 \\ -J_4 & -J_3 & J_1 \end{pmatrix}, \mathsf{J}_{31}^{\text{global}} = \begin{pmatrix} J_1 & -J_4 & -J_3 \\ J_4 & J_2 & -J_4 \\ -J_3 & J_4 & J_1 \end{pmatrix}. \tag{S4}$$

It is also a common (arguably much more prevalent) practice to write the spin Hamiltonian (S1) in the *local* coordinates, where the local z axes are defined along the local $[111]$ directions. The bases in the local coordinates of the four sublattices are defined as[31]

$$\hat{\mathsf{z}}_0 = \frac{1}{\sqrt{3}}(1,1,1), \hat{\mathsf{x}}_0 = \frac{1}{\sqrt{6}}(-2,1,1); \tag{S5a}$$
$$\hat{\mathsf{z}}_1 = \frac{1}{\sqrt{3}}(1,-1,-1), \hat{\mathsf{x}}_1 = \frac{1}{\sqrt{6}}(-2,-1,-1); \tag{S5b}$$
$$\hat{\mathsf{z}}_2 = \frac{1}{\sqrt{3}}(-1,1,-1), \hat{\mathsf{x}}_2 = \frac{1}{\sqrt{6}}(2,1,-1); \tag{S5c}$$
$$\hat{\mathsf{z}}_3 = \frac{1}{\sqrt{3}}(-1,-1,1), \hat{\mathsf{x}}_3 = \frac{1}{\sqrt{6}}(2,-1,1). \tag{S5d}$$

In the local coordinates, the spin Hamiltonian takes the form[31]

$$H = \sum_{ij} \left[J_{zz} \mathsf{S}_i^z \mathsf{S}_j^z - J_{\pm} \left(\mathsf{S}_i^+ \mathsf{S}_j^- + \mathsf{S}_i^- \mathsf{S}_j^+ \right) + J_{\pm\pm} \left(\gamma_{ij} \mathsf{S}_i^+ \mathsf{S}_j^+ + \gamma_{ij}^* \mathsf{S}_i^- \mathsf{S}_j^- \right) + J_{z\pm} \left(\zeta_{ij} \mathsf{S}_i^z \mathsf{S}_j^+ + \zeta_{ij}^* \mathsf{S}_i^z \mathsf{S}_j^- + i \longleftrightarrow j \right) \right], \tag{S6}$$

where γ_{ij} and ζ_{ij} are unimodular complex numbers,

$$\zeta = \begin{pmatrix} 0 & -1 & e^{i\pi/3} & e^{-i\pi/3} \\ -1 & 0 & e^{-i\pi/3} & e^{i\pi/3} \\ e^{i\pi/3} & e^{-i\pi/3} & 0 & -1 \\ e^{-i\pi/3} & e^{i\pi/3} & -1 & 1 \end{pmatrix}, \tag{S7a}$$

$$\gamma = -\zeta^*. \tag{S7b}$$

In the form (S6), the spin Hamiltonian has the advantage that when the spin flip interactions are negligible, i.e. in the limit $J_{\pm\pm} \longrightarrow 0$ and $J_{z\pm} \longrightarrow 0$, it reduces to a local XXZ model, which is studied in Refs.[6,10] and shown to support quantum spin liquid states.

To obtain the global exchange parameters in (S1) from the local exchange parameters in (S6), we just have to rotate the local bases $(\hat{\mathrm{x}}_s, \hat{\mathrm{y}}_s, \hat{\mathrm{z}}_s)$ such that they align with the global bases $(\hat{\mathbf{x}}, \hat{\mathbf{y}}, \hat{\mathbf{z}})$. Call these sublattice dependent rotations R_s. We then have, for example,

$$\mathsf{J}_{01}^{\text{global}} = \mathsf{R}_0^{-1} \mathsf{J}_{01}^{\text{local}} \mathsf{R}_1. \tag{S8}$$

The final result is

$$\begin{pmatrix} J_1 \\ J_2 \\ J_3 \\ J_4 \end{pmatrix} = \frac{1}{3} \begin{pmatrix} -1 & 4 & 2 & 2\sqrt{2} \\ 1 & -4 & 4 & 4\sqrt{2} \\ -1 & -2 & -4 & 2\sqrt{2} \\ -1 & -2 & 2 & -\sqrt{2} \end{pmatrix} \begin{pmatrix} J_{zz} \\ J_{\pm} \\ J_{\pm\pm} \\ J_{z\pm} \end{pmatrix}. \tag{S9}$$

The g tensor in global coordinates, which is sublattice dependent, can be obtained from that in local coordinates by rotations of the bases similar to the consideration in (S8). That is,

$$\mathsf{g}_s^{\text{global}} = \mathsf{R}_s^{-1} \mathsf{g}^{\text{local}} \mathsf{R}_s \tag{S10}$$

We list all the g tensors below for completeness[35].

$$\mathsf{g}_{s=0}^{\text{global}} = \begin{pmatrix} g_1 & g_2 & g_2 \\ g_2 & g_1 & g_2 \\ g_2 & g_2 & g_1 \end{pmatrix}, \mathsf{g}_{s=1}^{\text{global}} = \begin{pmatrix} g_1 & -g_2 & -g_2 \\ -g_2 & g_1 & g_2 \\ -g_2 & g_2 & g_1 \end{pmatrix}, \mathsf{g}_{s=2}^{\text{global}} = \begin{pmatrix} g_1 & -g_2 & g_2 \\ -g_2 & g_1 & -g_2 \\ g_2 & -g_2 & g_1 \end{pmatrix}, \mathsf{g}_{s=3}^{\text{global}} = \begin{pmatrix} g_1 & g_2 & -g_2 \\ g_2 & g_1 & -g_2 \\ -g_2 & -g_2 & g_1 \end{pmatrix}, \tag{S11}$$

where $g_1 = 2g_{xy}/3 + g_z/3$ and $g_2 = -g_{xy}/3 + g_z/3$.

S3 Mean Field Hamiltonian

On the one hand, the generic nearest neighbor $JK\Gamma$ model can be expressed solely in terms of the spinon pairing and hopping channels that signify the spin liquid phase,

$$H = \sum_{\lambda=x,y,z} \sum_{\langle ij \rangle \in \lambda} \left(H_{ij}^J + H_{ij}^K + H_{ij}^\Gamma \right) + \mu_3 \sum_{i\alpha} \left(f_{i\alpha}^\dagger f_{i\alpha} - 1 \right) + \left((\mu_1 + i\mu_2) \sum_i f_{i\downarrow} f_{i\uparrow} + \text{h.c.} \right); \tag{S12a}$$

$$H_{ij}^J = \begin{cases} -\frac{|J|}{4} \left(\hat{\mathbf{E}}_{ij}^\dagger \cdot \hat{\mathbf{E}}_{ij} + \hat{\mathbf{D}}_{ij}^\dagger \cdot \hat{\mathbf{D}}_{ij} \right), \text{for} J < 0; \\ -\frac{|J|}{4} \left(\hat{\chi}_{ij}^\dagger \hat{\chi}_{ij} + \hat{\Delta}_{ij}^\dagger \hat{\Delta}_{ij} \right), \text{for} J > 0; \end{cases} \tag{S12b}$$

$$H_{ij}^K = \begin{cases} -\frac{|K|}{8} \left(\hat{E}_{ij}^{\mu\dagger} \hat{E}_{ij}^\mu + \hat{E}_{ij}^{\nu\dagger} \hat{E}_{ij}^\nu + \hat{D}_{ij}^{\mu\dagger} \hat{D}_{ij}^\mu + \hat{D}_{ij}^{\nu\dagger} \hat{D}_{ij}^\nu \right), \text{for} K < 0; \\ -\frac{|K|}{8} \left(\hat{\chi}_{ij}^\dagger \hat{\chi}_{ij} + \hat{\Delta}_{ij}^\dagger \hat{\Delta}_{ij} + \hat{E}_{ij}^{\lambda\dagger} \hat{E}_{ij}^\lambda + \hat{D}_{ij}^{\lambda\dagger} \hat{D}_{ij}^\lambda \right), \text{for} K > 0; \end{cases} \tag{S12c}$$

$$H_{ij}^\Gamma = \begin{cases} -\frac{|\Gamma|}{8} \left(\left(\hat{E}_{ij}^\mu - \hat{E}_{ij}^\nu \right)^\dagger \left(\hat{E}_{ij}^\mu - \hat{E}_{ij}^\nu \right) + \left(\hat{D}_{ij}^\mu - \hat{D}_{ij}^\nu \right)^\dagger \left(\hat{D}_{ij}^\mu - \hat{D}_{ij}^\nu \right) + \hat{\chi}_{ij}^\dagger \hat{\chi}_{ij} + \hat{\Delta}_{ij}^\dagger \hat{\Delta}_{ij} + \hat{E}_{ij}^{\lambda\dagger} \hat{E}_{ij}^\lambda + \hat{D}_{ij}^{\lambda\dagger} \hat{D}_{ij}^\lambda \right), \text{for} \Gamma < 0; \\ -\frac{|\Gamma|}{8} \left(\left(\hat{E}_{ij}^\mu + \hat{E}_{ij}^\nu \right)^\dagger \left(\hat{E}_{ij}^\mu + \hat{E}_{ij}^\nu \right) + \left(\hat{D}_{ij}^\mu + \hat{D}_{ij}^\nu \right)^\dagger \left(\hat{D}_{ij}^\mu + \hat{D}_{ij}^\nu \right) + \hat{\chi}_{ij}^\dagger \hat{\chi}_{ij} + \hat{\Delta}_{ij}^\dagger \hat{\Delta}_{ij} + \hat{E}_{ij}^{\lambda\dagger} \hat{E}_{ij}^\lambda + \hat{D}_{ij}^{\lambda\dagger} \hat{D}_{ij}^\lambda \right), \text{for} \Gamma > 0. \end{cases} \tag{S12d}$$

The Lagrange multipliers $\mu_1, \mu_2, \mu_3 \in \mathbb{R}$ are introduced in (S12a) to enforce the single occupancy constraint (one spinon per site)

$$\sum_{\alpha} f_{i\alpha}^{\dagger} f_{i\alpha} = 1 \tag{S13}$$

on average. Note that we have carefully written the various interactions (S12b)-(S12d) in the form

$$H_{ij}^{X} = -|c^{X}| \sum_{O} \hat{O}_{ij}^{\dagger} \hat{O}_{ij}, \tag{S14}$$

from which a mean field decoupling naturally follows,

$$H_{ij}^{X,\mathrm{MF}} = -|c^{X}| \sum_{O} \left(O_{ij}^{*} \hat{O}_{ij} + O_{ij} \hat{O}_{ij}^{\dagger} - |O_{ij}|^2 \right), \tag{S15}$$

and the mean field energy is bounded from below (i.e. the stability requirement is satisfied). $\hat{O}_{ij}$ are the bond operators as before, while O_{ij} (without the hat) are variational parameters to minimize the mean field energy.

On the other hand, it is shown in Refs.[35,45], which provides a group theory analysis of the classical model (i.e. the spins are treated as three component vectors with fixed magnitude), that the nearest neighbor spin interactions on a tetrahedral unit in the pyrochlore lattice can be expressed as a summation of bilinears of the magnetic order parameters $\mathbf{m}_{\mathrm{X}}$, multiplied by some energy coefficients a_{X},

$$H^{\mathrm{tet}} = \frac{1}{2} \sum_{\mathrm{X}} a_{\mathrm{X}} |\mathbf{m}_{\mathrm{X}}|^2. \tag{S16}$$

Each $\mathbf{m}_{\mathrm{X}}$ is a linear combination of the components of the spins, while each a_{X} is a linear combination of the exchange couplings. The magnetic order parameters represent different $\mathbf{q} = \mathbf{0}$ classical spin configurations on the pyrochlore lattice. Since each unit cell contains one up and one down tetrahedra, the Hamiltonian is given by summing 2 times (S16) over the unit cells. As discussed in the main text, we keep only the FM ($\mathrm{X} = \mathrm{T}_{1,\mathrm{A}'}$) and AFM ($\mathrm{X} = \mathrm{E}$) order parameters (their expressions can be found in the main text) as they are the only relevant classical phases to $Yb_2Ti_2O_7$. Here we quote their respective energy coefficients,

$$a_{\mathrm{T}_{1,\mathrm{A}'}} = (2J_1 + J_2)\cos^2\theta_{\mathrm{T}_1} - (J_2 + J_3 - 2J_4)\sin^2\theta_{\mathrm{T}_1} + \sqrt{2}J_3 \sin 2\theta_{\mathrm{T}_1}, \tag{S17a}$$

$$a_{\mathrm{E}} = -2J_1 + J_2 + J_3 + 2J_4. \tag{S17b}$$

We represent the magnetic order parameters in terms of spinon operators, and carry out a mean field decoupling similar to (S15),

$$H^{\mathrm{MF}} = \sum_{\mathbf{R}} \sum_{X} a_{\mathrm{X}} \left(2\mathbf{m}_{\mathrm{X}} \cdot \hat{\mathbf{m}}_{\mathrm{X}} - |\mathbf{m}_{\mathrm{X}}|^2 \right), \tag{S18}$$

where $\mathbf{R}$ labels the unit cell (not individual site), and $\mathbf{m}_{\mathrm{X}}$ (without the hat) are now variational parameters. The stability requirement is satisfied as the coefficients $a_{\mathrm{T}_{1,\mathrm{A}'}}$ and a_{E} are negative in the $J_1 - J_2$ phase space (with $J_3 = -1$ fixed) under study.

The above formulation allows us to incorporate both the quantum spin liquid and magnetically ordered states into a single mean field Hamiltonian, with the introduction of a weighting factor $r \in [0, 1]$, such that the spin liquid component (involving $\chi_{ij}, \Delta_{ij}, \mathbf{E}_{ij}, \mathbf{D}_{ij}$) is multiplied by $1 - r$, while the magnetic order component (involving $\mathbf{m}_{\mathrm{T}_{1,\mathrm{A}'}}$ and $\mathbf{m}_{\mathrm{E}}$) by r.

S4 Details of the Spin Liquid Ansatzes

We discuss the $\mathbb{Z}_2$U and $U(1)$M spin liquid ansatzes in details, especially the interdependence of the spinon hopping and pairing parameters. The allowed forms of these mean field parameters are dictated by the symmetries of the system. Constraint arises when one symmetry element maps a bond to itself, or two different symmetry elements relates two different bonds. In this section, the term *mean field Hamitlonian* is referred specifically to as the Hamiltonian (S12a), which has only spin liquid channels, after the mean field decoupling.

S4.1 $\mathbb{Z}_2$ Uniform Ansatz

We first introduce the following 2×2 matrix whose components are the spinon creation and annihilation operators[30],

$$\Psi_i = \begin{pmatrix} f_{i\uparrow} & f_{i\downarrow} \\ f_{i\downarrow}^\dagger & -f_{i\uparrow}^\dagger \end{pmatrix}. \tag{S19}$$

The spin operator can then be expressed as

$$S_i^\mu = \frac{1}{4}\mathrm{Tr}\left(\Psi_i^\dagger \sigma^\mu \Psi_i\right), \tag{S20}$$

and the mean field Hamiltonian as

$$H^{\mathrm{MF}} = \sum_{ij}\sum_{\mu=0,x,y,z} \mathrm{Tr}\left(\sigma^\mu \Psi_i u_{ij}^\mu \Psi_j^\dagger\right), \tag{S21}$$

where u_{ij}^μ are 2×2 matrices of the mean field ansatzes. For instance, on the bond $\langle 01\rangle$, with the exchange couplings $J, K, \Gamma < 0$,

$$\begin{aligned}
u_{01}^0 &= \frac{|\Gamma|}{8}\begin{pmatrix} \chi_{01} & -\Delta_{01}^* \\ -\Delta_{01} & -\chi_{01}^* \end{pmatrix}, \\
u_{01}^x &= \frac{2|J|+|\Gamma|}{8}\begin{pmatrix} E_{01}^x & D_{01}^{x*} \\ -D_{01}^x & E_{01}^{x*} \end{pmatrix}, \\
u_{01}^y &= \frac{2|J|+|K|+|\Gamma|}{8}\begin{pmatrix} E_{01}^y & D_{01}^{y*} \\ -D_{01}^y & E_{01}^{y*} \end{pmatrix} - \frac{|\Gamma|}{8}\begin{pmatrix} E_{01}^z & D_{01}^{z*} \\ -D_{01}^z & E_{01}^{z*} \end{pmatrix}, \\
u_{01}^z &= \frac{2|J|+|K|+|\Gamma|}{8}\begin{pmatrix} E_{01}^z & D_{01}^{z*} \\ -D_{01}^z & E_{01}^{z*} \end{pmatrix} - \frac{|\Gamma|}{8}\begin{pmatrix} E_{01}^y & D_{01}^{y*} \\ -D_{01}^y & E_{01}^{y*} \end{pmatrix}.
\end{aligned}$$

We also have

$$u_{ii}^0 = \begin{pmatrix} \mu_3 & \mu_1 - i\mu_2 \\ \mu_1 + i\mu_2 & -\mu_3 \end{pmatrix} \tag{S23}$$

that enforces the single occupancy constraint (S13). In the form (S20), it is apparent that the spinon representation of spin is invariant under an $SU(2)$ gauge transformation

$$\Psi_i \longrightarrow \Psi_i G_i,\; G_i \in SU(2). \tag{S24}$$

We apply the symmetry operations passively, that is, transform the coordinate axes forward (equivalently transform the vectors backward)[46], such that

$$\mathbf{S}_i \xrightarrow{X} R_X^{-1}\mathbf{S}_{X(i)}, \tag{S25}$$

where X is an element of the space group and R_X is the $SU(2)$ spin rotation associated with X. In the representation (S20), the symmetry transformation (S25) is achieved by[30]

$$\Psi_i \xrightarrow{X} e^{i\sigma\cdot\hat{\mathbf{n}}\phi/2}\Psi_{X(i)}, \tag{S26}$$

where $\hat{\mathbf{n}}$ is a unit vector along the axis of rotation and ϕ is the angle of rotation associated with X. Therefore, X acts on the mean field Hamiltonian (S21) by

$$\begin{aligned}
H^{\mathrm{MF}} &\xrightarrow{X} \sum_{ij}\sum_{\mu=0,x,y,z}\mathrm{Tr}\left(e^{-i\sigma\cdot\hat{\mathbf{n}}\phi/2}\sigma^\mu e^{i\sigma\cdot\hat{\mathbf{n}}\phi/2}\Psi_{X(i)}u_{ij}^\mu\Psi_{X(j)}^\dagger\right) \\
&= \sum_{ij}\mathrm{Tr}\left(\Psi_{X(i)}u_{ij}^0\Psi_{X(j)}^\dagger\right) + \sum_{ij}\sum_{\mu=x,y,z}\mathrm{Tr}\left(\sum_{\nu=x,y,z} O_X^{-1\,\mu\nu}\sigma^\nu \Psi_{X(i)}u_{ij}^\mu\Psi_{X(j)}^\dagger\right),
\end{aligned} \tag{S27}$$

where the $SU(2)$ spin rotation R_X has been mapped to the $SO(3)$ rotation O_X on the Pauli matrices. The Hamiltonian should be left invariant under X by the definition of symmetry. Taking into account the $SU(2)$ gauge redundancy (S24), this implies that the mean field ansatzes should obey the relations

$$u_{X(i)X(j)}^0 = G_X(X(i))\,u_{ij}^0 G_X(X(j))^\dagger, \tag{S28a}$$

$$u_{X(i)X(j)}^{\mu=x,y,z} = \sum_{\nu=x,y,z} O_X^{\mu\nu} G_X(X(i))\,u_{ij}^\nu G_X(X(j))^\dagger, \tag{S28b}$$

where $G_X(i)$ is the $SU(2)$ gauge transformation associated with X at site i. To this end, we list the $SO(3)$ matrices O_X associated with some representative elements of the O_h point group discussed in Section S1,

$$O_{C_3^{[111]}} = \begin{pmatrix} 0 & 0 & 1 \\ 1 & 0 & 0 \\ 0 & 1 & 0 \end{pmatrix}, O_{C_2^x} = \begin{pmatrix} 1 & 0 & 0 \\ 0 & -1 & 0 \\ 0 & 0 & -1 \end{pmatrix}, O_{S_4^x} = \begin{pmatrix} 1 & 0 & 0 \\ 0 & 0 & -1 \\ 0 & 1 & 0 \end{pmatrix}, O_{\sigma_{\mathrm{d}}^{[011]}} = \begin{pmatrix} -1 & 0 & 0 \\ 0 & 0 & 1 \\ 0 & 1 & 0 \end{pmatrix}, O_{\mathscr{I}} = \begin{pmatrix} 1 & 0 & 0 \\ 0 & 1 & 0 \\ 0 & 0 & 1 \end{pmatrix}. \tag{S29}$$

All other space group elements can be constructed from these, e.g. $C_2^y = C_3^{[111]} C_2^x C_3^{[111]-1}$. The two fold rotation itself can be obtained by twice the four fold improper rotations, e.g. $C_2^x = (S_4^x)^2$. Note that, since spin is a pseudovector, it is invariant under inversion, hence the $SO(3)$ matrix associated with inversion is the identity. The reflections and improper rotations can be viewed as a combination of rotation and inversion, and their corresponding $SO(3)$ matrices only encode the rotation. For example, the reflection σ_{d} across the plane perpendicular to the $[011]$ direction is a rotation by π about the $[011]$ axis followed by inversion about the intersection of the axis and the plane.

On the other hand, time reversal $\mathscr{T}$ acts on the mean field Hamiltonian (S21) by

$$\begin{aligned} H^{\mathrm{MF}} &\xrightarrow{\mathscr{T}} \sum_{ij} \sum_{\mu=0,x,y,z} \mathrm{Tr}\left(-i\sigma^y \sigma^{\mu*} i\sigma^y \Psi_i u_{ij}^{\mu*} \Psi_j^\dagger\right) \\ &= \sum_{ij} \mathrm{Tr}\left(\Psi_i u_{ij}^{0*} \Psi_j^\dagger\right) + \sum_{ij} \sum_{\mu=x,y,z} \mathrm{Tr}\left(-\sigma^\mu \Psi_i u_{ij}^{\mu*} \Psi_j^\dagger\right). \end{aligned} \tag{S30}$$

Again, with the $SU(2)$ gauge redundancy, that $\mathscr{T}$ being a symmetry requires

$$u_{ij}^0 = G_{\mathscr{T}}(i) u_{ij}^{0*} G_{\mathscr{T}}(j)^\dagger, \tag{S31a}$$

$$u_{ij}^{\mu=x,y,z} = -G_{\mathscr{T}}(i) u_{ij}^{\mu*} G_{\mathscr{T}}(j)^\dagger. \tag{S31b}$$

Recall that the collection of the compound operators $G_X X$ (the symmetry group $\{X\}$ now includes both the space group elements and the time reversal) is known as the projective symmetry group (PSG), and we say that the symmetry X is realized projectively if G_X is nontrivial.

In the $\mathbb{Z}_2$U uniform ansatz, for every symmetry X of the system, we set the corresponding $SU(2)$ gauge transformation $G_X = 1$ to be trivial. We now investigate how the various symmetries limit the form of the spinon hopping and pairing parameters χ_{ij}, Δ_{ij}, $\mathbf{E}_{ij}$, and $\mathbf{D}_{ij}$. First, time reversal symmetry constrains the singlet parameters χ_{ij} and Δ_{ij} to be real, and the triplet parameters E_{ij}^μ and D_{ij}^μ to be imaginary, by (S31a) and (S31b). We also have $\mu_2 = 0$. Next, consider the bond $\langle 01 \rangle$, which is mapped to itself under C_2^x. By (S28a) and (S28b), we have $u_{10}^0 = u_{01}^0$, $u_{10}^x = u_{01}^x$, $u_{10}^y = -u_{01}^y$, and $u_{10}^z = -u_{01}^z$. As the singlet and triplet parameters obey the relations $\chi_{ji} = \chi_{ij}^*$, $\Delta_{ji} = \Delta_{ij}$, $E_{ji}^\mu = E_{ij}^{\mu*}$, $D_{ji}^\mu = -D_{ij}^\mu$, this implies $E_{01}^x = 0$ and $D_{01}^x = 0$. The bond $\langle 01 \rangle$ is also mapped to itself under the reflection $\sigma_{\mathrm{d}}^{[011]}$, which, similar to the analysis of the effect of C_2^x above, constrains $u_{01}^z = -u_{01}^y$, or $E_{01}^z = -E_{01}^y$ and $D_{01}^z = -D_{01}^y$. Finally, we can use C_3 or other symmetries to relate the mean field parameters on other bonds to those on $\langle 01 \rangle$. For the singlet parameters it is easy, $\chi_{ij} = \chi_{01}$ and $\Delta_{ij} = \Delta_{01}$ for all bonds $\langle ij \rangle$ by (S28a). For the triplet parameters, we give an example below,

$$\begin{pmatrix} u_{02}^x \\ u_{02}^y \\ u_{02}^z \end{pmatrix} = O_{C_3^{[111]}} \begin{pmatrix} u_{01}^x \\ u_{01}^y \\ u_{01}^z \end{pmatrix} = \begin{pmatrix} u_{01}^z \\ u_{01}^x \\ u_{01}^y \end{pmatrix}, \tag{S32}$$

or $E_{02}^x = -E_{01}^y$, $D_{02}^x = -D_{01}^y$, $E_{02}^y = 0$, $D_{02}^y = 0$, $E_{02}^z = E_{01}^y$, and $D_{02}^z = D_{01}^y$, by (S28b). The bond parameters on a down tetrahedron are the same as their counterparts on an up tetrahedron, i.e. $u_{ij\in\mathrm{down}}^\mu = u_{ij\in\mathrm{up}}^\mu$, by inversion symmetry. There is no further constraint from symmetries, and the number of independent mean field parameters χ_{01}, Δ_{01}, E_{01}^y, and D_{01}^y in the $\mathbb{Z}_2$U ansatz is four.

S4.2 $U(1)$ Monopole Flux Ansatz

The analysis of the $U(1)$M ansatz is in some way easier than that of the $\mathbb{Z}_2$U ansatz because the pairing terms Δ_{ij} and $\mathbf{D}_{ij}$ are zero. There is no need to introduce the matrix (S19) and write down the mean field Hamiltonian in the form (S21). We have instead

$$H^{\mathrm{MF}} = \sum_{ij} \sum_{\mu=0,x,y,z} u_{ij}^\mu \sum_{\alpha\beta} f_{i\alpha}^\dagger [\sigma^\mu]_{\alpha\beta} f_{j\beta} + \mathrm{h.c.}, \tag{S33}$$

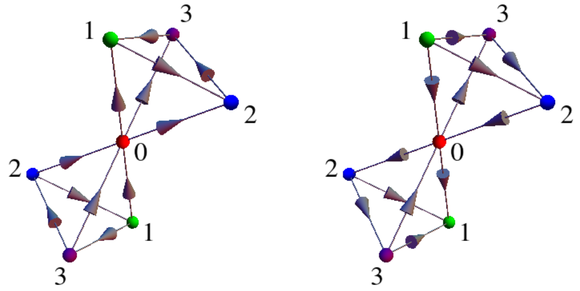

(a) (b)

Figure S3. (a) The configuration of link fields a_{ij}, which are the arguments of the singlet hopping parameters χ_{ij} (see (S37)), in the monopole flux ansatz. For each link connecting two sites i and j, a_{ij} is equal to $\pi/2$ ($-\pi/2$) along (against) the direction of the arrow. This gives a flux of $\pi/2$ on each elementary triangle. (b) The ansatz changes under a symmetry transformation X, for example $X = C_2^x$ as shown here. To restore the original configuration of link fields, we apply a sublattice dependent gauge transformation $G_X = \pm 1$, for example $G_{C_2^x}(0) = +1$, $G_{C_2^x}(1) = -1$, $G_{C_2^x}(2) = -1$, and $G_{C_2^x}(3) = +1$. The compound operators $G_X X$, which leave the mean field ansatz invariant, form the monopole flux PSG (see Table (S1)).

where u_{ij}^{μ} are now numbers that depend on the hopping terms instead of matrices. For instance, on the bond $\langle 01 \rangle$, with the exchange couplings $J, K, \Gamma < 0$,

$$
\begin{aligned}
u_{01}^{0} &= -\frac{|\Gamma|}{8}\chi_{01}^{*}, \\
u_{01}^{x} &= -\frac{|J|}{4}E_{01}^{x*} - \frac{|\Gamma|}{8}E_{01}^{x*}, \\
u_{01}^{y} &= -\frac{|J|}{4}E_{01}^{y*} - \frac{|K|}{8}E_{01}^{y*} - \frac{|\Gamma|}{8}\left(E_{01}^{y*} - E_{01}^{z*}\right), \\
u_{01}^{z} &= -\frac{|J|}{4}E_{01}^{z*} - \frac{|K|}{8}E_{01}^{z*} - \frac{|\Gamma|}{8}\left(E_{01}^{z*} - E_{01}^{y*}\right).
\end{aligned}
$$

In the spinon representation of spins, for an element X of the space group, the symmetry transformation (S25) is achieved by

$$
\begin{pmatrix} f_{i\uparrow} \\ f_{i\downarrow} \end{pmatrix} \xrightarrow{X} e^{i\sigma\cdot\hat{\mathbf{n}}\phi/2} \begin{pmatrix} f_{X(i)\uparrow} \\ f_{X(i)\downarrow} \end{pmatrix}, \tag{S35}
$$

where $\hat{\mathbf{n}}$ and ϕ are as defined previously. However, not all of the 48 elements of the full octahedral group O_h are respected in the $U(1)$M ansatz. The 24 elements that correspond to inversion, reflections (including glide symmetries), and improper rotations are broken, while the 24 elements that correspond to proper rotations (including screw symmetries) are realized within the simple PSG constructed in Ref.[34], where the site dependent gauge transformations $G_X = \pm 1$. Translational symmetry is also preserved. The proper rotations are[34], with the coordinate system defined in Fig. S2,

- e: the identity;
- $8\, C_3$: rotation by $\pm 2\pi/3$ about one of the local $[111]$ axes (the directions along the center to the corners of the tetrahedron);
- $3\, C_2$: rotation by π about one of the cubic axes (x, y and z directions);
- $6\, \widetilde{C}_4$: screw symmetry about one of the axes which are (i) parallel to x axis and going through $(0, a/4, 0)$, (ii) parallel to y axis and going through $(0, 0, a/4)$, and (iii) parallel to z axis and going through $(a/4, 0, 0)$ - rotation by $\mp\pi/2$ about one of these axes followed by translation by $a/4$ along that axis;
- $6\, \widetilde{C}_2$: screw symmetry about one of the edges (which connects two sublattices) of a tetrahedron - rotation by π about one of the edges followed by translation along that edge.

It is worth noting that the screw symmetries can be obtained by combining the improper rotations or the reflections with inversion, e.g. $\widetilde{C}_4^x = \mathscr{I} S_4^x$ and $\widetilde{C}_2^{\langle 01 \rangle} = \mathscr{I} \sigma_{\mathrm{d}}^{[011]}$. The $SO(3)$ matrices corresponding to the $SU(2)$ spin rotations generated by $\widetilde{C}_4^x$

Table S1. The projective symmetry group (PSG) of the monopole flux ansatz. The element $G_X X$ is denoted by X for simplicity, where X is one of the 24 proper rotations (including screw symmetries) of the O_h point group. The action of $G_X X$ is shown in (S39). The subscript s of the fermionic operator f_s indexes the sublattice.

e	$C_3^{[111]}$	$C_3^{[111]2}$	$C_3^{[1\bar{1}\bar{1}]}$	$C_3^{[1\bar{1}\bar{1}]2}$	$C_3^{[\bar{1}1\bar{1}]}$	$C_3^{[\bar{1}1\bar{1}]2}$	$C_3^{[\bar{1}\bar{1}1]}$	$C_3^{[\bar{1}\bar{1}1]2}$	C_2^x	C_2^y	C_2^z
f_0	f_0	f_0	f_3	$-f_2$	f_1	$-f_3$	f_2	$-f_1$	$-f_1$	$-f_2$	$-f_3$
f_1	f_2	f_3	f_1	f_1	$-f_3$	f_0	$-f_0$	$-f_2$	f_0	$-f_3$	f_2
f_2	f_3	f_1	$-f_0$	$-f_3$	f_2	f_2	$-f_1$	f_0	f_3	f_0	$-f_1$
f_3	f_1	f_2	$-f_2$	f_0	$-f_0$	$-f_1$	f_3	f_3	$-f_2$	f_1	f_0
$\widetilde{C}_4^x$	$\widetilde{C}_4^{x3}$	$\widetilde{C}_4^y$	$\widetilde{C}_4^{y3}$	$\widetilde{C}_4^z$	$\widetilde{C}_4^{z3}$	$\widetilde{C}_2^{\langle 01\rangle}$	$\widetilde{C}_2^{\langle 02\rangle}$	$\widetilde{C}_2^{\langle 03\rangle}$	$\widetilde{C}_2^{\langle 12\rangle}$	$\widetilde{C}_2^{\langle 23\rangle}$	$\widetilde{C}_2^{\langle 31\rangle}$
f_3	$-f_2$	f_1	$-f_3$	f_2	$-f_1$	f_1	f_2	f_3	$-f_0$	$-f_0$	$-f_0$
f_2	f_3	$-f_2$	$-f_0$	f_0	$-f_3$	f_0	f_1	$-f_1$	f_2	f_1	f_3
f_0	$-f_1$	f_3	f_1	$-f_3$	$-f_0$	$-f_2$	f_0	f_2	f_1	f_3	f_2
$-f_1$	$-f_0$	f_0	$-f_2$	f_1	f_2	f_3	$-f_3$	f_0	f_3	f_1	f_1

and $\widetilde{C}_2^{\langle 01\rangle}$ are

$$O_{\widetilde{C}_4^x} = \begin{pmatrix} 1 & 0 & 0 \\ 0 & 0 & 1 \\ 0 & -1 & 0 \end{pmatrix}, O_{\widetilde{C}_2^{\langle 01\rangle}} = \begin{pmatrix} -1 & 0 & 0 \\ 0 & 0 & 1 \\ 0 & 1 & 0 \end{pmatrix}. \tag{S36}$$

The monopole flux ansatz is first constructed for the nearest neighbor antiferromagnetic Heisenberg model on the pyrochlore lattice[34], where $u_{ij}^0 \sim |J|\chi_{ij}$ and $u_{ij}^{\mu=x,y,z} = 0$ in (S33). The singlet hopping parameter between two sites i and j takes the form

$$\chi_{ij} = \rho e^{ia_{ij}}, \rho > 0, a_{ij} = \pm\frac{\pi}{2}. \tag{S37}$$

The configuration of the link fields a_{ij} is visualized in Fig. S3a, such that along (against) the direction of the arrow from site i to j, a_{ij} equals to $+\pi/2$ $(-\pi/2)$. This gives a flux of

$$\sum_{\langle ij\rangle\in\triangle} a_{ij} = \frac{\pi}{2}, \tag{S38}$$

on each elementary triangle, if the orientation of the surface is chosen to be pointing towards the center of the tetrahedron. This can be thought of as a monopole of strength 2π sitting inside each tetrahedron, hence the name monopole flux state. Let X be any of the 24 symmetry elements. Then, for the mean field Hamiltonian of the AFM Heisenberg model,

$$\begin{aligned} H_{\text{HAFM}}^{\text{MF}} &\sim \sum_{ij} f_{i\alpha}^\dagger \chi_{ij} f_{j\alpha} \\ &\xrightarrow{X} \sum_{ij} f_{X(i)\alpha}^\dagger \chi_{ij} f_{X(j)\alpha} \\ &\xrightarrow{G_X} \sum_{ij} G_X(X(i))\, f_{X(i)\alpha}^\dagger \chi_{ij} f_{X(j)\alpha} G_X(X(j)), \end{aligned} \tag{S39}$$

where the site dependent gauge transformation $G_X = \pm 1$ is introduced to restore the ansatz (i.e. the original configuration of link fields),

$$G_X(X(i))\,\chi_{ij} G_X(X(j)) = \chi_{X(i)X(j)}. \tag{S40}$$

For example, under $C_3^{[111]}$, the ansatz remains the same as in Fig. S3a, so $G_{C_3^{[111]}}(s) = +1$ for all sublattices s. However, under C_2^x the configuration of link fields changes to that as in Fig. S3b, so we choose $G_{C_2^x}(0) = +1$, $G_{C_2^x}(1) = -1$, $G_{C_2^x}(2) = -1$, and $G_{C_2^x}(3) = +1$ to restore the original ansatz. To this end, we summarize the monopole flux PSG $\{G_X X\}$ for all the 24 symmetry elements X in Table S1, similar to Tables IV and V in Ref.[34]. The monopole flux ansatz is translationally invariant, i.e. it is the same for every physical unit cell of the pyrochlore lattice.

Table S2. Comparison between the local and global minima at Gaulin parametrization with the $\mathbb{Z}_2$U ansatz.

	local minimum			global minimum		
$B_z/\lvert J_3\rvert$	E	phase	S/S_0	E	phase	S/S_0
0	−0.439	SL	0	−0.442	FM*	0.975
0.002	−0.439	FM*	0.021	−0.454	FM*	0.996
0.004	−0.439	FM*	0.043	−0.465	FM	1
0.006	−0.440	FM*	0.066	−0.477	FM	1
0.008	−0.441	FM*	0.091	−0.489	FM	1
0.010	−0.442	FM*	0.122	−0.501	FM	1

Table S3. Comparison between the local and global minima at Coldea parametrization with the $\mathbb{Z}_2$U ansatz.

	local minimum			global minimum		
$B_z/\lvert J_3\rvert$	E	phase	S/S_0	E	phase	S/S_0
0	−0.471	SL	0	−0.481	AFM*	0.721
0.002	−0.471	FM*	0.017	−0.481	AFM*	0.726
0.004	−0.471	FM*	0.034	−0.483	cAFM*	0.736
0.006	−0.472	FM*	0.052	−0.485	cAFM*	0.756
0.008	−0.473	FM*	0.071	−0.489	cAFM*	0.791
0.010	−0.474	FM*	0.091	−0.495	FM*	0.952

Finally, we now extend the monopole flux ansatz to include the triplet hopping parameters, which appears in the mean field Hamiltonian of the nearest neighbor $JK\Gamma$ model on the pyrochlore lattice (S33), using the relation

$$u^{\mu}_{X(i)X(j)} = \sum_{\nu} G_X\left(X(i)\right) O_X^{\mu\nu} u^{\nu}_{ij} G_X\left(X(j)\right), \tag{S41}$$

which can be derived in a way similar to (S27). The $SO(3)$ matrices O_X of some representative symmetry elements X can be found in (S29) and (S36).

Since inversion symmetry is broken, the bond parameters of the up and down tetrahedra no longer obey $u^{\mu}_{ij\in\mathrm{up}} = u^{\mu}_{ij\in\mathrm{down}}$ as in the $\mathbb{Z}_2$U ansatz. We define v^{μ}_{ij} as u^{μ}_{ij} for the bond $\langle ij\rangle$ on a down tetrahedron. The form of the singlet hopping parameter χ_{ij} has already been fixed by (S37). For the triplet hopping parameters on the bond $\langle 01\rangle$, C_2^x constrains $u^x_{10} = -u^x_{01}$, $u^y_{10} = u^y_{01}$, and $u^z_{10} = u^z_{01}$ by (S41), which implies E^x_{01} is imaginary, while E^y_{01} and E^z_{01} are real. u^{μ}_{ij} on other bonds are related to u^{μ}_{01} by C_3, while v^{μ}_{ij} are related to u^{μ}_{ij} by $\widetilde{C}_4$ or $\widetilde{C}_2$. The number of independent mean field parameters is four. There is no further constraint from symmetries. In the absence of pairing channel, for a free fermion hopping Hamiltonian like (S33) at zero temperature, the single occupancy constraint is satisfied (on average) by half filling of the momentum states, so there is no need to introduce extra Lagrange multipliers (though μ_3 is often identified with the Fermi level in the literature).

S5 Comparisons between the Local and Global Minima from the Mean Field Self Consistent Calculations

We tabulate the energy per unit cell E, the phase, and the reduction of magnetic order parameter in magnitude relative to its classical value S/S_0 (see main text), of the local and global minima, which correspond to a pure spin liquid/spin liquid dominant and pure magnetic order/magnetic order dominant phases respectively, at various magnetic field strength B_z, for the $\mathbb{Z}_2$U ansatz, at Gaulin and Coldea parametrizations (see Tables S2 and S3). A representative value $r = 0.23$ of the weighting factor is chosen. $S/S_0 \longrightarrow 0$ indicates that the magnetic order is very weak and the system is highly quantum, while $S/S_0 \longrightarrow 1$ indicates that the system approaches the classical limit. In other words, the ratio S/S_0 is a good indicator of the quantumness of the system. As B_z increases, the energy difference between the local and global minima grows more significant. Once B_z exceeds $\sim 0.01|J_3|$, the spin liquid dominant solution becomes so unfavorable that the self consistent calculations always yield the completely magnetic solution. Similar comparisons are made for the $U(1)$M ansatz in Tables S4 and S5.

S6 Properties of the Spinon Band Structure in the Pure Magnetically Ordered States

We explain three aspects of the spinon band structure in the pure magnetic phase (where the magnetic order parameters are finite and the spin liquid parameters are zero): (i) flatness (dispersionless), (ii) symmetry about zero energy, and (iii) four fold

Table S4. Comparison between the local and global minima at Gaulin parametrization with the $U(1)$M ansatz.

	local minimum			global minimum		
$B_z/\|J_3\|$	E	phase	S/S_0	E	phase	S/S_0
0	−0.474	FM*	0.008	same as left		
0.002	−0.474	FM*	0.039	same as left		
0.004	−0.475	FM*	0.072	same as left		
0.006	−0.476	FM*	0.110	−0.477	FM	1
0.008	−0.478	FM*	0.161	−0.489	FM	1
0.010	−0.480	FM*	0.210	−0.501	FM	1

Table S5. Comparison between the local and global minima at Coldea parametrization with the $U(1)$M ansatz.

	local minimum			global minimum		
$B_z/\|J_3\|$	E	phase	S/S_0	E	phase	S/S_0
0	−0.509	AFM*	0.009	same as left		
0.002	−0.509	FM*	0.031	same as left		
0.004	−0.510	FM*	0.056	same as left		
0.006	−0.511	FM*	0.083	same as left		
0.008	−0.512	FM*	0.113	same as left		
0.010	−0.514	FM*	0.152	same as left		
0.015	−0.520	FM*	0.247	−0.525	FM	1

degeneracy. Recall that the magnetic order parameters are linear combinations of the spin components, so that the mean field Hamiltonian (S18) takes the form

$$\begin{aligned} H_{\mathrm{MO}}^{\mathrm{MF}} &= \sum_{\mathbf{R}} \sum_{s\in\mathbf{R}} \begin{pmatrix} f_{\mathbf{R},s,\uparrow}^{\dagger} & f_{\mathbf{R},s,\downarrow}^{\dagger} \end{pmatrix} \left(c_s^x \sigma^x + c_s^y \sigma^y + c_s^z \sigma^z\right) \begin{pmatrix} f_{\mathbf{R},s,\uparrow} \\ f_{\mathbf{R},s,\downarrow} \end{pmatrix} \\ &= \sum_{\mathbf{k}} \sum_{s=0,1,2,3} \begin{pmatrix} f_{\mathbf{k},s,\uparrow}^{\dagger} & f_{\mathbf{k},s,\downarrow}^{\dagger} \end{pmatrix} \left(\sum_{\mu=x,y,z} c_s^{\mu} \sigma^{\mu} \right) \begin{pmatrix} f_{\mathbf{k},s,\uparrow} \\ f_{\mathbf{k},s,\downarrow} \end{pmatrix} \end{aligned} \tag{S42}$$

with the coefficients $c_s^{\mu} \in \mathbb{R}$. Since neither spinon hopping nor pairing at two different sites is present, Fourier transform does not introduce any nontrivial phase factor $e^{i\mathbf{k}\cdot(\mathbf{R}_i-\mathbf{R}_j)}$ in the second equality of (S42). This explains the flatness of the spinon bands as the energy eigenvalues are independent of the momentum **k**. Furthermore, written in the basis $(f_{\mathbf{k},0,\uparrow}, f_{\mathbf{k},0,\downarrow}, \cdots, f_{\mathbf{k},3,\uparrow}, f_{\mathbf{k},3,\downarrow})$, the Hamiltonian matrix is an 8×8 block matrix whose nonzero blocks are the four 2×2 matrices along the diagonal. Diagonalization yields the energy eigenvalues

$$\omega_{\mathbf{k}s} = \pm\sqrt{(c_s^x)^2 + (c_s^y)^2 + (c_s^z)^2}. \tag{S43}$$

The $\pm$ sign means that the spinon bands are symmetric about the zero level. Finally, from (S43) we see that the energy eigenvalues depend on the coefficients c_s^{μ} only through the second power. We examine the FM and AFM order parameters and find that their respective set of coefficients c_s^{μ} satisfies $c_s^{\mu} = \pm c_{s'}^{\mu}$ for different sublattices s and s'. This implies the four fold degeneracy.
